# Vascular Response of Tetrabromobisphenol a in Rat Aorta: Calcium Channels Inhibition and Potassium Channels Activation

**DOI:** 10.3390/toxics10090529

**Published:** 2022-09-09

**Authors:** Joana Feiteiro, Sandra M. Rocha, Melissa Mariana, Cláudio J. Maia, Elisa Cairrao

**Affiliations:** 1 CICS-UBI-Health Sciences Research Centre, University of Beira Interior, 6200-506 Covilhã, Portugal; 2 FCS-UBI, Faculty of Health Sciences, University of Beira Interior, 6200-506 Covilhã, Portugal

**Keywords:** tetrabromobisphenol A, relaxation, calcium channels, potassium channels, rat aortic, A7r5 cells

## Abstract

Tetrabromobisphenol A (TBBPA) is a flame retardant widely used to reduce flammability. It is an endocrine disruptor, and due to constant human exposure, some concerns have been raised regarding its impact on human health. Studies showed that TBBPA affects oxidative stress, cell proliferation and intracellular calcium levels. However, the vascular consequences of TBBPA exposure are still relatively unexplored. Hence, this work aimed to analyse TBBPA effects on rat aortic smooth muscle and its action mechanisms. Through an ex vivo approach, Wistar rat aortas were used in an organ bath to evaluate the vascular effect of TBBPA (0.01–100 μM). Additionally, TBBPA’s mode of action was studied through calcium and potassium channel inhibitors. Resorting to in vitro studies, A7r5 cells were used to analyse L-Type voltage-gated calcium channel (VGCC) activity through the whole-cell configuration of the patch clamp technique, and the mRNA expression of proteins and ion channels involved in vascular contractility. The results showed vasorelaxation of rat aorta induced by TBBPA exposure, involving the inactivation of L-Type VGCC and activation of potassium channels, and the modulation of mRNA expression of L-type calcium and large-conductance calcium 1.1 and the BK_Ca_ 1.1 α- and β_1_ -subunit channels, soluble guanylyl cyclase and protein Kinase G.

## 1. Introduction

One of the most prevalent brominated flame retardants is tetrabromobisphenol A (TBBPA or 2,6-Dibromo-4-[2-(3,5-dibromo-4-hydroxyphenyl)propan-2yl]phenol), used in plastic, textile and paper [1]. This compound is considered an endocrine disruptor and it has been already found in food, dust, water, air, soil, and consequently, in animals and humans [1]. 

So far, most relevant studies have been performed in animals, mainly in rodents, showing an association between TBBPA exposure and reproductive, developmental, and neurobehavioral effects [2,3,4]. Additionally, it was also demonstrated that this compound can alter thyroid hormone levels [5,6], and has been related to nephrotoxicity, hepatotoxicity and carcinogenicity [7,8,9]. Specifically, TBBPA leads to an increase of intracellular calcium levels ([Ca^2+^]_i_) and to cell death in cerebellar granule cells’ primary neurons [10]. Moreover, in rat pancreatic β-cells, TBBPA can also affect some parameters of oxidative stress, namely nitric oxide (NO) and intracellular reactive oxygen species (ROS), and mitochondrial superoxide levels [11]. These results showed that the disruption of calcium (Ca^2+^) homeostasis is involved in the formation of ROS and cell death, mechanisms in which TBBPA is involved. 

It is known that vascular tone is regulated by [Ca^2+^]_i_, which is the main factor of vascular smooth muscle cells’ (SMC) contractility, and NO [12,13]. In rat small mesenteric arteries and rabbit cerebral arteries, it is suggested that NO may activate large-conductance Ca^2+^-activated K^+^ (BK_Ca_) channels in a cyclic guanosine monophosphate (cGMP)-independent manner, and may modulate the frequency of Ca^2+^ sparks affecting the activity of BK_Ca_ channels [14,15].

Given these observations, it is important to analyse how TBBPA exposure affects the vascular tonus and to understand the mechanistic pathways underlying these effects. Thus, the aim of this work was to assess the effects of TBBPA in rat aortic smooth muscle and to investigate its potential signalling pathway. For that purpose, the TBBPA effect on contracted endothelium-denuded rat aorta was analysed by ex vivo organ bath experiments. Using A7r5 cells, TBBPA effects on voltage-dependent Ca^2+^ current (I_Ca,L_) were analysed through the whole cell configuration of the patch clamp technique, and after 24 h TBBPA exposure, the expression of potassium (K^+^) and Ca^2+^ channels, soluble guanylate cyclase (sGC) and cGMP-dependent protein kinase (PKG) were evaluated. 

## 2. Methods

### 2.1. Drugs and Chemicals

The drugs used in cell culture and contractility, MTT and electrophysiology experiments were purchased from Sigma-Aldrich Chemistry (Sintra, Portugal). The reagents for RT-qPCR technique were bought NZYTech (Lisboa, Portugal), and Grisp (Porto, Portugal). In addition, the bovine serum albumin (BSA) was purchased from Fisher Scientific and the foetal bovine serum (FBS) from Biochrom (Cambrige, United Kingdom). The stock solutions of Phenylephrine (Phenyl), Noradrenaline (NA), tetraethylammonium (TEA) and 4-aminopyridine (4-AP) were made in distilled water, while TBBPA, Nifedipine (Nif), glybenclamide (Gly) and Bay-K 8644 were dissolved in absolute ethanol and stored at −20 °C. Appropriate dilutions were prepared before the experiments in each specific solution, Krebs’ solution to be used in organ bath, electrophysiology external solution in patch clamp technique and FBS-free culture medium in MTT assay and RT-qPCR technique. The final concentrations of ethanol (vehicle) did not exceed 0.1%.

### 2.2. Ex Vivo Studies 

#### Contractility Experiments in Isolated Rat Thoracic Aorta Rings

The use of male adult Wistar rats (Charles-River, Barcelona, Spain) was approved by the Animal Research Committee of University of Beira Interior (CICS-UBI, Covilhã, Portugal) and follows the regulations of the European Convention for the Protection of Vertebrate Animals Used for Experimental and Other Scientific Purposes (Directive 2010/63/EU). The procedures for euthanasia, thoracotomy and organ bath technique were performed as previously performed by our group [16,17]. After confirming the absence of endothelium using acetylcholine (1 μM), the rat aorta rings were contracted with Phenyl (1 μM), NA (1 μM) and isosmotic KCl (60 mM) solution. Upon each contraction, the effects of TBBPA (0.01–100 μM) were evaluated. Additionally, to analyse the influence of Ca^2+^ and K^+^ channels, different inhibitors were added:

- Nif (0.001 and 1 μM), an inhibitor of voltage-gated calcium channels (VGCC);
- TEA (1000 μM), an inhibitor of conductance Ca^2+^-activated K^+^ (BK_Ca_) channel;
- 4-AP (1000 μM), an inhibitor of voltage-gated potassium channels (K_v_);
- Gly (10 μM), an inhibitor of ATP-sensitive potassium (K_ATP_).

In these experiments, before contraction, the rat aorta rings were incubated 15 min with these K^+^ channel inhibitors and the effects of TBBPA and Nif in the presence of these drugs were analysed. Ethanol was used as control at the same percentage used to dissolve TBBPA. Each experiment was conducted in several rat aorta rings from at least five different rats.

### 2.3. In Vitro Studies

#### 2.3.1. Culture of A7r5 Cells 

The A7r5 cell line is a commercial vascular smooth muscle cell line obtained from embryonic rat aorta (Sigma-Aldrich, Portugal) and a suitable model to study the contractile function, mainly the calcium homeostasis. The culture of the cells was performed according to Mariana et al. [16]. After confluence, the cells were maintained in a culture medium without FBS for 24 h. Before each experiment, the cells were trypsinized using a commercial trypsin-EDTA solution (0.025%). These cells were used to perform MTT assay, electrophysiology and real-time quantitative polymerase chain reaction (RT-qPCR) experiments. 

#### 2.3.2. Cell Viability 

A7r5 cells’ viability and proliferation in response to TBBPA exposure were measured using the MTT assay. This assay was performed according to the methodology described by Feiteiro et al. [18]. Confluent cells were treated for 24 h with different concentrations of TBBPA (0.01, 0.1, 1, 10, 30, 50, 100, 500 and 1000 μM), after which, 200 μL MTT solution (0.5 mg/mL) were added. After 4 h (37 °C, 5% CO_2_ and 95% of humidity) of exposure to MTT, it was removed, and the formazan crystals were dissolved in DMSO and converted into a purple colour, indicating the amount of formazan production. Colour intensity was measured at 570 nm using a photometer (EZ Read 400, Microplate Reader, Biochrom). 

#### 2.3.3. Electrophysiology Experiments

For the electrophysiological experiments, the cells were kept at 4 °C in medium without FBS until the initiation of the experience. To analyse the L-type VGCC current (I_Ca,L_), the patch clamp technique was used in the whole-cell configuration, as described by Cairrao et al. [19] and Mariana et al. [16]. Different concentrations of TBBPA (0.01, 1, 10, 50 and 100 μM) dissolved in external solution, were studied in basal and BAY K8644-stimulated (0.01 μM) I_Ca,L_.

#### 2.3.4. Real-Time Quantitative Polymerase Chain Reaction (RT-qPCR) 

RT-qPCR was the technique used to assess the mRNA expression of L-type calcium channel a1C-subunit (Cav1.2), BK_Ca_ 1.1 α- and β_1_- subunits (BK_Ca_ 1.1α and BK_Ca_ β_1_), sGC (Gucci_α_) and protein kinase cGMP-dependent 1 α-subunit (PRKG 1α) in response to TBBPA after 24 h of treatment. This procedure was performed according to Feiteiro et al. [18]. For these cells, cyclophilin A (Cyc A) was used as internal control to normalize gene expression. The efficiency of the amplification (CFX Connect; Real-Time System; Bio- Rad, Hercules, CA, USA) was defined for all primer sets using serial dilutions of cDNA samples (1:1, 1:5 and 1:25). For the qPCR, a 20 μL reaction was prepared (1 μL cDNA, 0.3 μM of each primer, except for L-type (0.4 μM), and 10 μL SYBR Green/Fluorescein qPCR Master Mix). Then, initial denaturation at 95 °C for 5 min was followed by 40 cycles of 95 °C (10 s), 60 °C was the annealing temperature (30 s) and then at 72 °C (10 s). The amplified PCR fragments (60 °C to 95 °C at a rate of 0.05 °C/s) were verified by melting curve analysis. All samples were run in triplicate for each qPCR assay. The normalized mRNA expression value was calculated following the mathematical model proposed by Pfaffl using the formula 2^−ΔΔCt^ [20]. All oligonucleotide primers are indicated in Table 1.

### 2.4. Statistical Analysis

According to the number and type of the variables tested in this work, different and specific statistical methods were applied. For cell viability, a comparison between different concentrations of TBBPA and control was analysed using a one-way analysis of variance (ANOVA) followed by Dunnett’s post hoc test to determine significant differences among the means. In the contractility experiments, statistical significance between two groups was analysed using Student’s *t*-test, while a comparison between different concentrations of TBBPA was analysed using one-way ANOVA followed by Tukey post hoc test. Additionally, to analyse the statistical difference of the effect of Nif and K+ channel inhibitors on the effect of TBBPA, the Two-way ANOVA with interaction followed by the Holm–Sidak post-hoc tests were applied. In the electrophysiology experiments, comparison between different concentrations of TBBPA was analysed using one-way ANOVA followed by Tukey post hoc test to determine significant differences. Finally, to evaluate the statistical differences in the mRNA expression one-way ANOVA followed by Dunnett’s multiple comparison test was used. 

In all cases the statistical significance was considered for a *p*-value lower than 0.05 and all values are presented as the mean ± SD (standard deviation) of number of experiments. Software Origin 8.5.1. was used for the graphic design and SigmaStat Statistical Analysis System version 4.0 (2016) (San Jose, CA, USA) for data analysis. 

## 3. Results

This research study assessed how TBBPA affects rat aorta smooth muscle. The TBBPA-mediated vascular effects were analysed a functional (through the organ bath and patch-clamp) and genomic levels (24 h exposure of TBBPA in A7r5 cells). The design of this study was chosen in accordance with previous works performed by our research group [16,17,21,22], in which bisphenol A (BPA), an analogue of TBBPA, was one of the compounds analysed in those studies. These authors demonstrated that this experimental design is adequate to analyse how these hazardous compounds impair vascular function. A wide range of concentrations is appropriate to study the toxic effects of environmental contaminants, so in this study, we used 0.01–1000 μM of TBBPA, which is in accordance with a recent study [18] that demonstrated that only the highest concentrations decreased the viability of human umbilical smooth muscle cells.

### 3.1. Ex Vivo Studies 

#### 3.1.1. Effects of TBBPA on Isolated Rat Aorta

To evaluate the effects of TBBPA on rat aorta contractility, after removing the endothelium, the aortic rings were first exposed to Phenyl (1 μM), NA (1 μM) and KCl (60 mM) solution. The maximum contractions promoted by these three contractile agents were 2.02 ± 0.7 g, 2.01 ± 0.7 g and 2.01 ± 0.6 g, respectively, and were not statistically significant (*p* = 0.997). However, when TBBPA was added to the resting tension, the results showed that none of the TBBPA concentrations (0.01–100 μM) affected the basal tension (data not shown).

**Figure 1:**
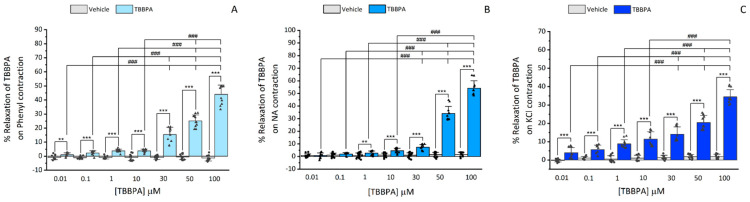
TBBPA vasorelaxation (0.01–100 μM) in rat aorta rings contracted with (**A**) Phenylephrine (Phenyl, 1 μM, number of rat aortas = 7), (**B**) Noradrenaline (NA, 1 μM, number of rat aortas = 7) and (**C**) KCl (60 mM, number of rat aortas = 6). Results are expressed as a percentage (%) of relaxation on contractility. Each bar represents the mean values, the vertical lines the SD and the dots and the triangles the replicates for each n. * Represents significant statistical differences between each TBBPA concentrations and the respective vehicle (** *p* < 0.01 and *** *p* < 0.001, Student’s *t*-test) and ### represents statistical differences between all TBBPA concentrations (*p* < 0.001, one-way ANOVA followed by Tukey’s post hoc tests).

**Figure 2:**
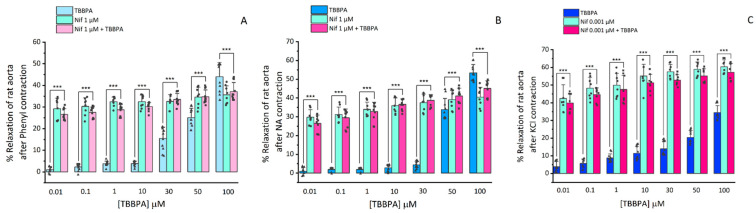
Relaxation of rat aortas with TBBPA (0.01–100 μM), Nif (0.1 and 1 μM) and Nif plus TBBPA upon contraction with (**A**) Phenylephrine (Phenyl, 1 μM, number of rat aortas = 8), (**B**) Noradrenaline (NA, 1 μM, number of rat aortas = 8) and (**C**) KCl (60 mM, number of rat aortas = 8). Results are expressed as a percentage (%) of relaxation on contractility. Each bar represents the mean values, the vertical lines the SD and the dots, squares and triangles the replicates for each n. *** Represents significant statistical differences between TBBPA and Nif + TBBPA: *** *p* < 0.001. Data were analysed using two-way ANOVA followed by Holm–Sidak post-hoc test.

Afterwards, the TBBPA effects were analysed over the contractions of the Phenyl (1 μM), NA (1 μM) and KCl (60 mM). As displayed in Figure 1, TBBPA provoked a concentration-dependent relaxation over the precontracted aortic rings, either with Phenyl (Figure 2A), NA (Figure 2B) or KCl (Figure 2C), with a maximum effect at 100 μM (the highest concentration). The relaxations elicited by this concentration of TBBPA on Phenyl- NA- or KCl-contracted rat aorta were 44 ± 6.3%, 53.35 ± 4.23% and 34.43 ± 3.8%, respectively. In Phenyl and NA-contracted rat aorta, the relaxant effects of TBBPA are more prominent, indicating that the contractile agent used can modulate the TBBPA response. Also in Figure 1, it is shown that the vehicle did not have significant effects on contracted arteries in any of the concentrations used.

#### 3.1.2. Influence of L-type Ca^2+^ Channels on TBBPA-Induced Vasorelaxation on Isolated Rat Aorta

To analyse the involvement of L-type VGCC in the TBBPA vasorelaxant effect, after contraction with Phenyl (1 μM), NA (1 μM) and isosmotic KCl (60 mM) solution, the arteries were exposed to Nif (0.001 and 1 μM) and the TBBPA (0.01–100 μM). Isosmotic KCl solution induces contraction by extracellular Ca^2+^ influx, through depolarisation and opening of L-type VGCC. Nif, being a specific blocker of these channels, when added at a concentration of 1 μM, leads to a relaxation of almost 100% (data not shown) Thus, a lower concentration (0.001 μM) had to be used in KCl-contracted arteries to better analyse the effect of the TBBPA plus Nif. Figure 2 shows that Nif caused vasorelaxation in all Phenyl-, NA- and KCl-contracted arteries with a statistically significant interaction between the different concentrations of TBBPA and the effect triggered by Nif treatment (*p* ≤ 0.001). Concerning the rat aorta contracted with Phenyl and NA (Figure 2A,B), the vasorelaxation induced by the combined application Nif and TBBPA (0.01–50 μM) was significantly higher than the individual TBBPA effect (*p* < 0.001). However, at 100 μM, the vasorelaxation induced by Nif plus TBBPA was lower than the individual TBBPA effect (*p* < 0.001). While in KCl-contracted arteries (Figure 2C), the vasorelaxation induced by Nif plus TBBPA was significantly higher than the individual TBBPA effect in all concentrations (*p* < 0.001). The vasorelaxation induced by Nif plus TBBPA was similar to the individual effect of Nif (*p* > 0.05) in all applied conditions and TBBPA concentrations. Regardless of the contractile agent, the inactivation of L-type VGCC seems to be involved in the TBBPA vasorelaxant effect. 

#### 3.1.3. Influence of K^+^ Channels on TBBPA-Induced Vasorelaxation on Isolated Rat Aorta

**Figure 3:**
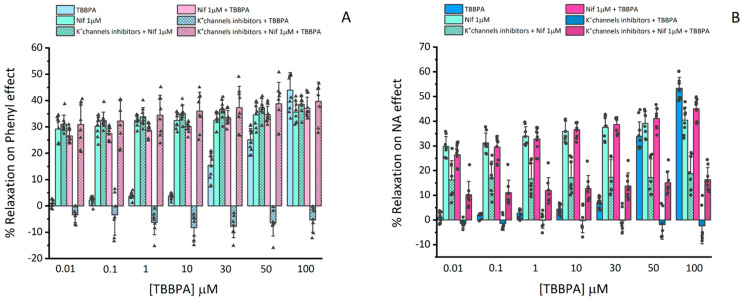
Vasorelaxant effects of TBBPA (0.01–100 μM), nifedipine (Nif, 1 μM), K+ channel inhibitors (TEA, 1000 μM); 4-4-AP, 1000 μM and Gly, 10 μM), with Nif, Nif with TBBPA, and K+ channel inhibitors with Nif and TBBPA on rat aorta rings contracted with (**A**) Phenylephrine (Phenyl, 1 μM, number of rat aortas = 7) and (**B**) Noradrenaline (NA, 1 μM, number of rat aortas = 7). Each bar represents the mean values, the vertical lines the SD and the dots the replicates for each n.

Considering the previous results, the following approach was to analyse the involvement of K^+^ channels in the vasorelaxation mechanism induced by TBBPA in rat aorta. Thus, TEA, 4-AP and Gly were used as different K^+^ channel inhibitors. Firstly, before contraction, rat aorta rings were exposed for 15 min to TEA (1000 μM), 4-AP (1000 μM) and Gly (10 μM), and we observed that these inhibitors did not cause a significant effect on Phenyl and NA (1 μM) contraction (data not shown). After that, the Nif (1 μM) effect and cumulative concentrations of TBBPA (0.01–100 μM) were analysed, as shown in Figure 3. A statistical interaction was observed between the TBBPA levels, and the effect triggered by Nif plus TEA, 4-AP and Gly treatment for Phenyl and NA contractions (*p* ≤ 0.001). 

The following results for Phenyl contracted arteries will be summarized in Figure 3A and the statistics represented in Table 2, in which TBBPA will be called *Ct1*, Nif of *Ct2* and K^+^ inhibitors plus Nif of *Ct3*. The obtained results showed that the synergetic effect of Nif and TBBPA was significantly higher than *Ct1* effect at 0.01–50 μM of TBBPA (*p* < 0.001), but at 100 μM of TBBPA, the combined application of Nif and TBBPA was significantly lower than *Ct1* (*p* < 0.001). Moreover, the effects induced by K+ channel inhibitors with TBBPA were significantly lower than *Ct1* at 0.01–100 μM, and the combined application of K+ channel inhibitors, Nif and TBBPA was significantly higher than *Ct1* (0.01–100 μM). The *Ct2* effect was not statistically significant with either Nif plus TBBPA or with the effect of joint application of K+ channel inhibitors, Nif and TBBPA (*p* > 0.05). However, the effect of *Ct2* was significantly higher compared to the effect induced by the K+ channel inhibitors with TBBPA (*p* < 0.001). In addition, the effect of application of *Ct3* at 0.01, 0.1, 1 and 10 μM of TBBPA was significantly higher than the effect induced by Nif plus TBBPA (*p* < 0.05), while the effect induced by K+ channel inhibitors with TBBPA was significantly lower than *Ct3* (*p* < 0.001). However, the *Ct3* effect was not significantly different compared to the effect of the combined application of K+ channel inhibitors, Nif and TBBPA (*p* > 0.05).

In the same way as Phenyl, the results and statistics related to NA will be presented in Figure 3B and in Table 3, with the same designations *Ct1*, *Ct2* and *Ct3*. The results showed that combined application of Nif and TBBPA was significantly higher than *Ct1* at 0.01–50 μM of TBBPA (*p* < 0.001). However, at 100 μM the effect was significantly lower than *Ct1* (*p* < 0.001). The effect of K+ channel inhibitors plus TBBPA was significantly lower than *Ct1* at the three highest TBBPA concentrations (30, 50 and 100 μM) (*p* < 0.01 and *p* < 0.001), as well as the combined application of K+ channel inhibitors, Nif and TBBPA at 0.01, 1, 10 and 50 (*p* < 0.001) and 30 μM (*p* < 0.01), which was also significantly lower than the effect of *Ct1* (*p* < 0.001). On the contrary, at 100 μM concentrations of TBBPA, the effect of *Ct1* was significantly higher than the combined application of K+ channel inhibitors, Nif and TBBPA (*p* < 0.001). Regarding *Ct2*, the effect induced by K+ channel inhibitors plus TBBPA and combined applications of K+ channel inhibitors, Nif and TBBPA were significantly lower than the vasorelaxation caused by *Ct2* (*p* < 0.001) at all concentrations of TBBPA. The effect of *CT3* was significantly lower than the combined application of Nif and TBBPA (*p* < 0.001), and higher when compared to the combined applications of K+ channel inhibitors and TBBPA (*p* < 0.001). Furthermore, the *Ct3* effect was only significantly higher for TBBPA at 0.01 and 0.1 μM of TBBPA than the combined applications of K+ channel inhibitors, Nif and TBBPA (*p* < 0.05). 

### 3.2. In Vitro Studies 

#### 3.2.1. Assessment of Viability (MTT Assay) 

**Figure 4:**
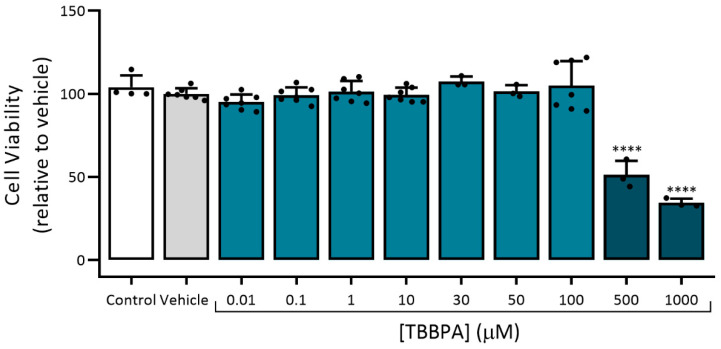
A7r5 cell viability under the effect of TBBPA. Data are expressed as a percentage (%) of cellular viability. Each bar represents the mean values and vertical lines the SD and the dots the replicates for each n. **** Represents statistical differences between TBBPA and Vehicle (*p* < 0.0001, one-way ANOVA followed by Dunnett’s post hoc test).

The cellular viability of A7r5 cells exposed to TBBPA (0.01, 0.1, 1, 10, 30, 50, 100, 500 and 1000 μM) was analysed using MTT assay. Besides TBBPA, the cells were exposed to the culture medium as control, and to ethanol, the solvent used to dissolve TBBPA (vehicle), for 24 h. As shown in Figure 4, TBBPA at 500 and 1000 μM significantly decreased the cell viability (*p* < 0.0001). Thus, in all the experiments, the TBBPA concentrations used were 0.01, 0.1, 1, 10, 30, 50 and 100 μM.

#### 3.2.2. Effects of TBBPA on I_Ca,L_ in A7r5 Cells

**Figure 5:**
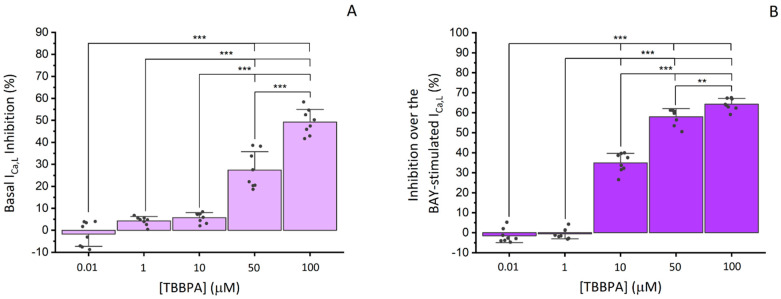
Effects of TBBPA (0.01–100 μM) on I_Ca,L_ in A7r5 Cells. (**A**) Inhibitory effects of TBBPA on basal I_Ca,L_ expressed in percent variation over the amplitude of basal I_Ca,L_ (**B**) inhibitory effects of TBBPA on the I_Ca,L_ stimulated by BAY (0.01 μM), expressed in percent variation over the amplitude of BAY-stimulated I_Ca,L_. Each bar represents the mean values, the vertical lines the SD and the dots the replicates for each n. * Represents statistical differences between TBBPA concentrations: ** *p* < 0.01, *** *p* < 0.001. Data were analysed using two-way ANOVA followed by Holm–Sidak post-hoc test.

In A7r5 cells, L-type VGCC current (I_Ca,L_) was analysed through the whole-cell patch clamp technique. Figure 5A showed the effect of the TBBPA concentrations used in this study on I_Ca,L_ of which 50 and 100 μM significantly inhibited basal I_Ca,L_ (*p* < 0.001). This inhibition was reversible after washout. The I_Ca,L_ density mean value was −1.82 ± 1.26 pA/pF (n = 33).

In order to analyse TBBPA effect on stimulated I_Ca,L_, we resorted to BAY K8644 (0.01 μM), as a potent and specific activator of the L-type VGCC, which stimulated the calcium current by 60.40 ± 30.22% above the basal level. Considering that BAY effects returned to the initial levels after washout we confirmed that the current analysed was I_Ca,L_. Figure 5B summarizes the effects of TBBPA on the calcium current, in which a significant inhibition was observed for concentrations of 10, 50 and 100 μM (*p* < 0.01 and *p* < 0.001), with maximum values of 34.96 ± 4.45%, 57.95 ± 3.80%, and 64.24 ± 2.72 % respectively. The vehicle used to dissolve TBBPA, ethanol, did not affect basal or stimulated I_Ca,L_ (−1.8 ± 2.75%) (data not shown). 

#### 3.2.3. Effects of TBBPA on the Expression of Cav1.2, BK_Ca_ β_1_, BK_Ca_ 1.1α, Gucci_α_ and PRKG 1α

**Figure 6:**
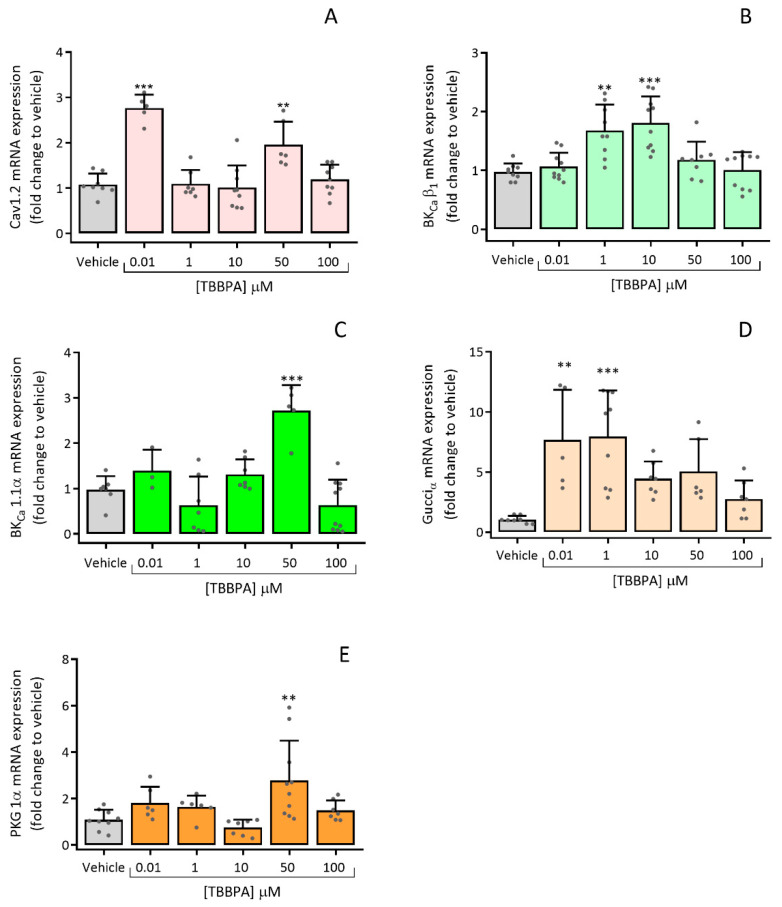
Relative expression in A7r5 cells exposed to TBBPA (0.01–100 μM) (24 h) (**A**) Cav1.2 channels, (**B**) BK_Ca_ β_1_ subunit channels, (**C**) BK_Ca_ 1.1α subunit channels, (**D**) sGC (Gucci_α_) and (**E**) PRKG 1α subunit. Cyc A was used as a housekeeping gene to normalize the mRNA expression. Each bar represents the mean values and vertical lines the SD of four independent experiments performed in triplicate and the dots the replicates for each n. * Represents significant statistical differences in comparison to vehicle (control): ** *p* < 0.01, *** *p* < 0.001. Data were analysed using ANOVA followed by Dunnett’s multiple comparison test.

The expression of ion channels and proteins implicated in the regulation of vascular tone, interfering with the contraction and relaxation mechanisms of the SMC, were evaluated using RT-qPCR. The effects of TBBPA on mRNA expression of channels’ subunits (Cav1.2, BK_Ca_ β_1_ and BK_Ca_ 1.1α) and proteins (Gucci_α_ and PRKG 1α) are represented in Figure 6. 

Concerning the ion channels the mRNA expression was significantly increased when A7r5 cells were incubated with TBBPA 0.01 and 50 μM for Cav1.2 channels (*p* < 0.001 and *p* < 0.05, Figure 6A), TBBPA 1 and 10 μM for BK_Ca_ β_1_ subunit (*p* < 0.01 and *p* < 0.001 respectively, Figure 6B) and TBBPA 50 μM for BK_Ca_ 1.1α subunit (*p* < 0.001, Figure 6C). Similarly, mRNA expression of Gucci_α_, was significantly higher for TBBPA 0.01 e 1 μM (*p* < 0.01 and *p* < 0.001, respectively, Figure 6D), while for PRKG 1α it was for TBBPA 50 μM (*p* < 0.01, Figure 6E). All these results were compared to the vehicle (control group). 

## 4. Discussion

TBBPA’s endocrine-disrupting properties are currently under European Chemicals Agency assessment [23]. However, according to the harmonized classification (European Union), TBBPA is already considered extremely toxic to aquatic life, and this effect can be long lasting [24]. 

The adverse effects of TBBPA in Ex vivo and In vitro models have been demonstrated in several studies. Recently, it was demonstrated that TBBPA induces vasorelaxation of the human umbilical artery (HUA) involving the NO/sGC/cGMP/PKG pathway and the influx of calcium, suggesting that TBBPA exposure modifies HUA vascular homeostasis [18]. In order to provide a clarification of these results, there was a need to analyse TBBPA effects in rat aorta, since this sample is considered a universal model at the vascular level. Furthermore, studies carried out in the rat aorta using a TBBPA analogue considered to be more toxic at the vascular level, the BPA, showed that it inhibits the calcium channels [17].

Experimental studies using animal models can offer a quicker and more flexible approach for the study of TBBPA effects on human health, and can contribute to the discovery of the mechanism of action and dose–response characteristics [1]. ECHA has also identified TBBPA as a suspected cause of cancer, since it has been classified as category 1B (Carc1B) based on animal studies. Most of those studies considered TBBPA as a carcinogenic compound in rodents, with possible mechanisms being disruption of oestrogen homeostasis and thyroid hormone pathway, oxidative stress, inflammation and immunosuppression [23]. In addition, TBBPA also leads to an increase in intracellular [Ca^2+^] and affects NO, ROS and mitochondrial superoxide levels [10,11,25]. As already mentioned, the intracellular [Ca^2+^] and NO are vital for vascular tonus regulation, and considering that TBBPA affects these parameters, the main goal of this work was to understand the real impact of TBBPA at a vascular level. For that purpose, ex vivo and in vitro experiments on rat aorta were performed. Regarding these arteries, the vascular contractility was analysed, and using cultured A7r5 cells, it was possible to (1) observe the TBBPA effects in the Ca^2+^ current, and (2) analyse the mRNA expression levels of Cav1.2, BK_Ca_ β_1_, BK_Ca_ 1.1α, Gucciα and PRKG 1α after 24 h exposure of TBBPA. 

Several studies using zebrafish, fathead minnow, rainbow trout, *Eisenia fetida* and earthworm species, showed that TBBPA can induce chronic toxicity after 14 and 96 h exposure [26,27,28,29]. However, in rodents, TBBPA induces small acute toxicity and has low availability [30]. Therefore, by analysing the A7r5 cells viability, it was found that after exposure to 500 and 1000 μM TBBPA, there was a decrease in cell viability of 66 and 49%, respectively. These results indicate that high concentrations of TBBPA have toxic effects on vascular smooth muscle cells. Considering these results, we excluded the concentrations 500 and 1000 μM in all the experiments. 

Through the ex vivo studies, it was possible to show for the first time that TBBPA induces relaxation on rat aorta rings pre-contracted either with Phenyl, NA or KCl (60 mM). We can suggest that, in addition to having a dose-dependent effect, it is not NO production-dependent, since the arteries are devoid of endothelium. Thus, TBBPA was uniquely responsible for the relaxation effect. It should be noted that the vasorelaxation induced by TBBPA was less pronounced in arteries contracted with KCl than those contracted with Phenyl or NA, which can be explained by the different vascular pathways of these three contractile agents. NA mediates vascular contractility through the activation of the α1A-, α1B-, α1D-, β1-, and β2-adrenoceptors [31,32], which act differently. The α1- adrenoceptors are coupled to the Gq protein activation, leading to an increase of [Ca^2+^]_i_ from the sarcoplasmic reticulum and causing contraction [33,34,35]. On the other hand, β1- and β2-adrenoceptors will lead to vasorelaxation. These receptors are coupled to Gs protein, inducing the activation of adenylate cyclase and increasing the intracellular cAMP concentration [36,37]. Also associated with the NA contraction is the influx of extracellular Ca^2+^ via voltage- or receptor-operated Ca^2+^ channels [38]. In the case of Phenyl, it causes contraction by binding only to α1-adrenoreceptors. As described above, these adrenoreceptors are associated with Gq protein, causing the increase in [Ca^2+^]_i_, which results in contraction [35,39,40]. Considering these results, TBBPA effects may be mediated by α1-, β1-, and β2-adrenoceptors’ receptor activation and by ion channels involved in the vascular response, namely Ca^2+^ and K^+^ channels. Regarding the KCl contraction, it is mainly triggered by membrane depolarization, and consequently, by the opening of VGCC, mainly L-Type, promoting the entry of extracellular Ca^2+^ [16,17]. Our results suggest that the decrease of Ca^2+^ influx by blockage of L-type VGCC is the main pathway involved in the TBBPA vasorelaxation. 

The L-type VGCC are the main Ca^2+^ channels responsible for the contractile or relaxant effects [12,41]. Thus, to understand the involvement of TBBPA on Ca^2+^ influx by ion channels, Nif, a specific inhibitor of L-type VGCC, was used on the contracted endothelium-denuded rat aorta rings, followed by TBBPA. The results demonstrated that Nif causes relaxation in rat aorta precontracted with Phenyl (1 μM), NA (1 μM) and KCl (60 mM). Even using a lower concentration of Nif (0.001 μM) in the arteries contracted with KCl, the induced relaxation was superior to that induced by Nif (1 μM) in the arteries contracted by Phenyl and NA, which proves that the KCl contraction is due to the opening of the L-type VGCC. 

From the results regarding the effects of TBBPA, it was clear that the vasorelaxation induced by the combined exposure to Nif and TBBPA was significantly higher than the individual TBBPA effect, but identical to the individual Nif effect, suggesting that TBBPA may share the same mechanism as Nif, or act by an interrelated pathway involving the inactivation of L-type VGCC. Despite the fact that there are no significant differences, in contractions with Phenyl and NA, the individual effect of TBBPA at 100 μM was higher than the individual Nif effect, demonstrating that K^+^ channels may also be involved in the TBBPA mechanism. The K^+^ channels contribute to relaxation in vascular smooth muscle by repolarization of the plasma membrane, leading to the closure of L-type VGCC channels [42]. 

Considering the possible involvement of K^+^ channels, the next approach was to analyse the influence of these channels on vasorelaxation induced by TBBPA on isolated rat aorta. For the different K+ channel inhibitors, TEA (inhibitor of BK_Ca_ channels, 1000 μM), 4-4-AP (inhibitor of K_v_ channels 1000 μM) and Gly (inhibitor of K_ATP_ channels, 10 μM) were used prior to Phenyl and NA contractions. Concerning the arteries contracted with Phenyl, our results demonstrated that the individual TBBPA effect was higher than the combined application of K+ channel inhibitors and TBBPA, and it was lower than the effect triggered by K+ channel inhibitors, Nif and TBBPA; moreover, the combined application of K+ channel inhibitors and Nif was higher than the effect of the K+ channel inhibitors plus TBBPA. These results suggest that TEA, 4-AP and Gly clearly inhibit the TBBPA effect, modifying the vasorelaxant response of this compound in rat aorta. These results show that activation of K^+^ channels is another pathway involved in vasorelaxation induced by TBBPA, whose increment of cGMP leads to the activation of PKG, and consequently, to a decrease in [Ca^2+^]_i_ [17,42]. The results obtained for rat aorta rings contracted with NA demonstrate that TBBPA individual effect (30, 50 and 100 μM) was higher than K+ channel inhibitors plus TBBPA, and at all concentrations, it was lower than the combined application of K+ channel inhibitors, Nif and TBBPA. Additionally, the relaxation induced by K+ channel inhibitors with TBBPA and by the combined applications of K+ channel inhibitors, Nif and TBBPA were significantly lower than vasorelaxation caused by the individual Nif effect. Overall, in arteries contracted with NA, the K^+^ inhibitors also modify the vasorelaxant response of TBBPA in a concentration-dependent manner. These data indicate that TBBPA vasorelaxation may occur either by inactivation of L-type VGCC channels or by activation of K^+^ channels, mainly through BK_Ca_, K_v_ and K_ATP_.

Taking into account the ex vivo results obtained, to confirm if the mechanisms of TBBPA-induced vasorelaxation were L-type VGCC dependent, patch clamp experiments in A7r5 cells were performed. In fact, TBBPA can inhibit L-type VGCC activity in vascular smooth muscle cells from rat aorta, with more notable effects at 50 and 100 μM, corresponding to 27.44 and 49.21% of inhibition, respectively. Furthermore, it also inhibits BAY-stimulated I_Ca,L_, with the maximum effect observed for the same concentrations (57.95 and 64.24%, respectively), confirming the TBBPA inhibitory effect on I_Ca,L_. These results are in accordance with a previous study performed by our research group, in which bisphenol A (BPA), which is an analogue of TBBPA, leads to vasorelaxation of rat aorta by inhibition of the L-type VGCC [17].

Regarding the TBBPA effects on the expression of these channels, the results showed an increase in mRNA expression levels of Cav1.2 channels in A7r5 cells increased after exposure to TBBPA, suggesting that this compound may interfere with Ca^2+^ homeostasis. These findings are in accordance with a study performed by Reistad et. al, in which rat cerebellar granule cells exposed to TBBPA increased the [Ca^2+^]_i_ and reduced the NMDA receptor antagonists, increasing cell death [10]. As mentioned previously, besides the inhibition of Ca^2+^ channels, the vasorelaxation induced by TBBPA may also be due to an increase in the K^+^ channels’ activity. Based on this proposition, the mRNA expression levels of BK_Ca_ 1.1 α- and β_1_- subunits were also analysed, showing an increased response to TBBPA incubation. Similarly, the mRNA expression levels of sGC (Gucci_α_) and PRKG 1α subunits were also increased when compared to control, indicating that TBBPA modulates these proteins´ expression. 

These are promising findings, which may lead to a better understanding of TBBPA vascular toxicity and the methods by which it affects human health. Thus far, few studies have related TBBPA exposure with cardiac health. When evaluating TBBPA cardiac developmental toxicity in zebrafish embryos and larvae, it was found that this compound led to ROS production and cardiomyocyte apoptosis [43], and to cardiac and blood circulation system impairment [44]. On the other hand, also available in the literature are several studies regarding the in vivo effects of BPA, demonstrating that it can lead to cardiovascular toxic events, including hypertension, arrhythmias, atherosclerosis, cardiac ischaemia and anomalies [45,46,47,48,49,50,51]. Considering that BPA, being an analogue of TBBPA, is involved in several cardiac complications, further in vivo studies are needed to demonstrate whether TBBPA affects the cardiovascular system the same way, and whether the in vitro and ex vivo effects revealed in this study could lead to possible diseases.

## 5. Conclusions

In this work, we demonstrated, for the first time, that TBBPA induces vasorelaxation of the rat aorta. Specifically, we were able to discover part of the action mechanism of TBBPA, which may be interrelated with the pathway involving L-type VGCC inactivation, or even share the same action mechanism as Nif. However, the presence of K^+^ inhibitors modified the effect of TBBPA, which leads us to conclude that the activation of K^+^ channels may be another pathway involved in TBBPA vasorelaxation. These data were corroborated by the results obtained in the analysis of mRNA expression, in which it was observed that TBBPA modulates the expression of proteins and ion channels involved in vascular contractility. Overall, these remarks suggest that TBBPA exposure interferes with vascular homeostasis through Ca^2+^ and K^+^ channels. 

## Figures and Tables

**Table 1:** Oligonucleotide primers used for real-time polymerase chain reaction.

Gene	GenBank Accession No.	Primer (5′-3′)	Concentration (μM)
Cyc A	NM_017090.2	Fw: 5- CAA GAC TGA GTG GCT GGA TGG -3 Rv: 5- GCC CGC AAG TCA AAG AAA TTA GAG -3	0.3
Cav1.2	NM_012517.2	Fw: 5- CTC GAA GTT GGG AGA ACA GC -3 Rv: 5- GAC GAA ACC CAC GAA GAT GT -3	0.4
BK_Ca_ β_1_	NM_019273.1	Fw: 5- CCA GGA ATC CAC CTG TCA CT -3 Rv: 5- TCA CAT CAA CCA AGG CTG TC -3	0.3
BK_Ca_ 1.1α	NM_031828.1	Fw: 5- GTC TGC ATC TTT GGG GAT GT -3 Rv: 5- GGG GAA GTT GTG CAG TGT TT -3	0.3
Gucci_α_	NM_017090.2	Fw: 5- GTG TGC CTC GGA AAA TCA AT -3 Rv: 5- ATC TCG GGG TGA ACA CAA AG -3	0.3
PRKG 1α	NM_001105731.3	Fw: 5- CGT GAG GCT ATA CCG GAC AT -3 Rv: 5- GCA AAC GCT TCT ACC ACA CA -3	0.3

**Table 2:** Statistical differences in the relaxation of rat aortas contracted with Phenyl (1 μM) in different conditions. *Ct1* represents the individual TBBPA effect; *Ct2* represents the individual Nif effect; *Ct3* represents the potassium channels inhibitors with Nif. * Represents significant statistical differences between different controls and other conditions: * *p* < 0.05, *** *p* < 0.001, n.s. represents no significant differences (*p* > 0.05). Data were analysed using two-way ANOVA followed by Holm–Sidak post-hoc test.

				[TBBPA] μM						
				0.01	0.1	1	10	30	50	100
Contraction with Phenylephrine (1 μM)	TBBPA (*Ct1*)	VS	Nif 1 μM + TBBPA	***	***	***	***	***	***	***
			K^+^ channels inhibitors + TBBPA	*	*	***	***	***	***	***
			K^+^ channels inhibitors + Nif 1 μM + TBBPA	***	***	***	***	***	***	*
	Nif 1 μM (*Ct2*)	VS	Nif 1 μM + TBBPA	n.s	n.s	n.s	n.s	n.s	n.s	n.s
			K^+^ channels inhibitors + TBBPA	***	***	***	***	***	***	***
			K^+^ channels inhibitors + Nif 1 μM + TBBPA	n.s	n.s	n.s	n.s	n.s	n.s	n.s
	K^+^ channels inhibitors + Nif 1 μM (*Ct3*)	VS	Nif 1 μM + TBBPA	*	*	*	*	n.s	n.s	n.s
			K^+^ channels inhibitors + TBBPA	***	***	***	***	***	***	***
			K^+^ channels inhibitors + Nif 1 μM + TBBPA	n.s	n.s	n.s	n.s	n.s	n.s	n.s

**Table 3:** Statistical differences in the relaxation of rat aortas contracted with NA (1 μM) in different conditions. *Ct1* represents the individual TBBPA effect; *Ct2* represents the individual Nif effect; *Ct3* represents the potassium channels inhibitors with Nif. * Represents significant statistical differences between different controls and other conditions: * *p* < 0.05, ** *p* < 0.01, *** *p* < 0.001, n.s. represents no significant differences (*p* > 0.05). Data were analysed using two-way ANOVA followed by Holm–Sidak post-hoc test.

				[TBBPA] μM						
				0.01	0.1	1	10	30	50	100
Contraction with Noradrenaline (1 μM)	TBBPA (*Ct1*)	VS	Nif 1 μM + TBBPA	***	***	***	***	***	***	***
			K^+^ channels blockage + TBBPA	n.s	n.s	n.s	n.s	**	***	***
			K^+^ channels blockage + Nif 1 μM + TBBPA	***	***	***	***	**	***	***
	Nif 1 μM (*Ct2*)	VS	Nif 1 μM + TBBPA	n.s	n.s	n.s	n.s	n.s	n.s	n.s
			K^+^ channels blockage + TBBPA	***	***	***	***	***	***	***
			K^+^ channels blockage + Nif 1 μM + TBBPA	***	***	***	***	***	***	***
	K^+^ channels + Nif 1 μM (*Ct3*)	VS	Nif 1 μM + TBBPA	***	***	***	***	***	***	***
			K^+^ channels blockage + TBBPA	***	***	***	***	***	***	***
			K^+^ channels blockage + Nif 1 μM + TBBPA	*	*	n.s	n.s	n.s	n.s	n.s

## Data Availability

Not applicable.
